# Rapid Intrahepatic Progression of Hepatocellular Carcinoma after Transarterial Chemoembolization: A Case Report

**DOI:** 10.7759/cureus.5305

**Published:** 2019-08-02

**Authors:** Tanveer H Siraj, Asim Tameez Ud Din, Farooq Mohyud Din Chaudhary, Sultan Ahmad, Khaleeq H Siddiqui

**Affiliations:** 1 Gastroenterology, Nishtar Medical University & Hospital, Multan, PAK; 2 Internal Medicine, Rawalpindi Medical University, Rawalpindi, PAK; 3 Internal Medicine, NewYork-Presbyterian Queens, Flushing, USA

**Keywords:** hepatocellular carcinoma, transarterial chemoembolization, acute on chronic liver failure

## Abstract

The prevalence of hepatocellular carcinoma (HCC) is increasing worldwide. Multiple strategies are available for its management including surgical removal, chemotherapeutic drugs, and ablative and chemoembolization procedures. Transarterial chemoembolization (TACE) is currently being used for the unresectable intrahepatic tumor with no vascular invasion or metastasis to other organs. The common adverse effects associated with this technique involve self-limiting fever, vomiting, and abdominal pain. Liver failure is reported in a few cases. In this report, we present a case of 37-year-old HCC patient who experienced rapid progression of tumor following TACE. Our patient came to the Gastroenterology & Hepatology Department, Nishtar Hospital, Multan, with signs concerning acute liver failure within a few months following TACE. On triphasic computed tomography (CT) scan, there was evidence of multiple new infiltrating lesions in both lobes of the liver and portal vein thrombosis, which were not present before TACE procedure. Hence, we made a diagnosis of acute, chronic liver disease due to the rapid intrahepatic progression of HCC. This is a rare side effect of TACE procedure and highlights the significance of proper counseling of the patients undergoing this intervention.

## Introduction

Hepatocellular carcinoma (HCC) is one of the most prevalent cancers all around the world. Most of the patients are diagnosed at the terminal stage when limited options of curative treatment are available. Multiple procedures are available for its management including surgery, chemoembolization, ablation, and chemotherapy. This largely depends on the stage of the tumor and the overall status of the patient. Transarterial chemoembolization (TACE) is considered a good therapeutic option for unresectable tumors which are not metastasized or involved the blood vessels [1]. The common adverse effects associated with TACE in these subsets of patients are reported in a prospective observational study. It mainly comprises self-limiting symptoms including fever, gastrointestinal features like vomiting and pain in the abdomen. Few cases of acute liver failure and deaths are also reported in the study [2]. Rapid recurrence and progression of HCC is a rare occurrence. Herein, we present a case of a 37-year-old HCC patient who developed a rapid progression of his underlying tumor after receiving TACE.

## Case presentation

A 37-year-old male patient presented to the Gastroenterology & Hepatology Department, Nishtar Hospital, Multan, Pakistan in July 2019, with complaints of jaundice and abdominal distention from the last two weeks.

The patient had a history of chronic hepatitis B (CHB) infection diagnosed since 2008. At that time, he had elevated levels of alanine aminotransferase (ALT), detectable hepatitis B virus (HBV) DNA levels, and no evidence of cirrhosis. He was advised tablet Tenofovir 300 mg once daily. However, the patient took treatment with poor compliance and was lost to follow-up.

**Figure 1:**
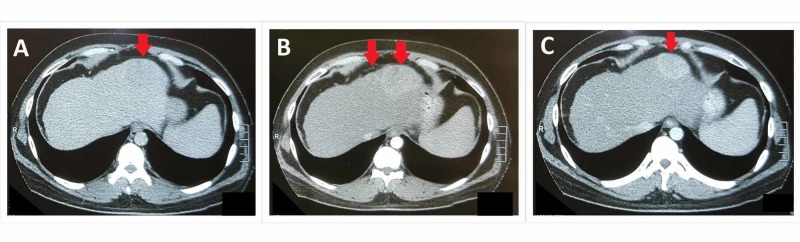
Pre-TACE Triphasic CT abdomen showing plain (A), arterial (B), and venous (C) phases of study There are two lobulated arterially enhancing (B) lesions (larger lesion measures 8.5 x 5.1 x 5.3 cm, smaller lesion measures 1.5 x 1.2 cm) in the left lobe of liver showing washout on the venous phase (C). The liver has irregular margins. CT: computed tomography; TACE: transarterial chemoembolization

In March 2019, the patient reported to a physician with the complaints of fatigability, body aches, loss of appetite, and weight loss for the past few weeks. He also had started drinking alcohol for the past few years and was now consuming alcohol on a daily basis. There was no history of illicit drug use. Family history was non-significant with respect to the liver and metabolic diseases. Examination findings included yellow sclera, mildly enlarged tender liver and splenomegaly with no evidence of ascites or peripheral edema. The patient had detectable HBV DNA levels and raised alpha-fetoprotein (AFP) levels. Further workup revealed two arterially enhancing lesions (larger one being 8.5 cm and the smaller one 1.5 cm in size) in the left lobe of liver on Triphasic Computed Tomography (CT) of the abdomen (Figure 1) consistent with hepatocellular carcinoma (HCC). The liver had an irregular surface. Portal vein measured 1.4 cm without evidence of thrombosis. There were no ascites and no evidence of metastasis. The patient was diagnosed with HCC (Barcelona clinic liver cancer [BCLC] stage B, child class B, performance status 0), CHB, and alcohol-related liver disease. As the patient's HCC was outside the resectability and transplant criteria, he was referred for transarterial chemoembolization (TACE). 

**Figure 2:**
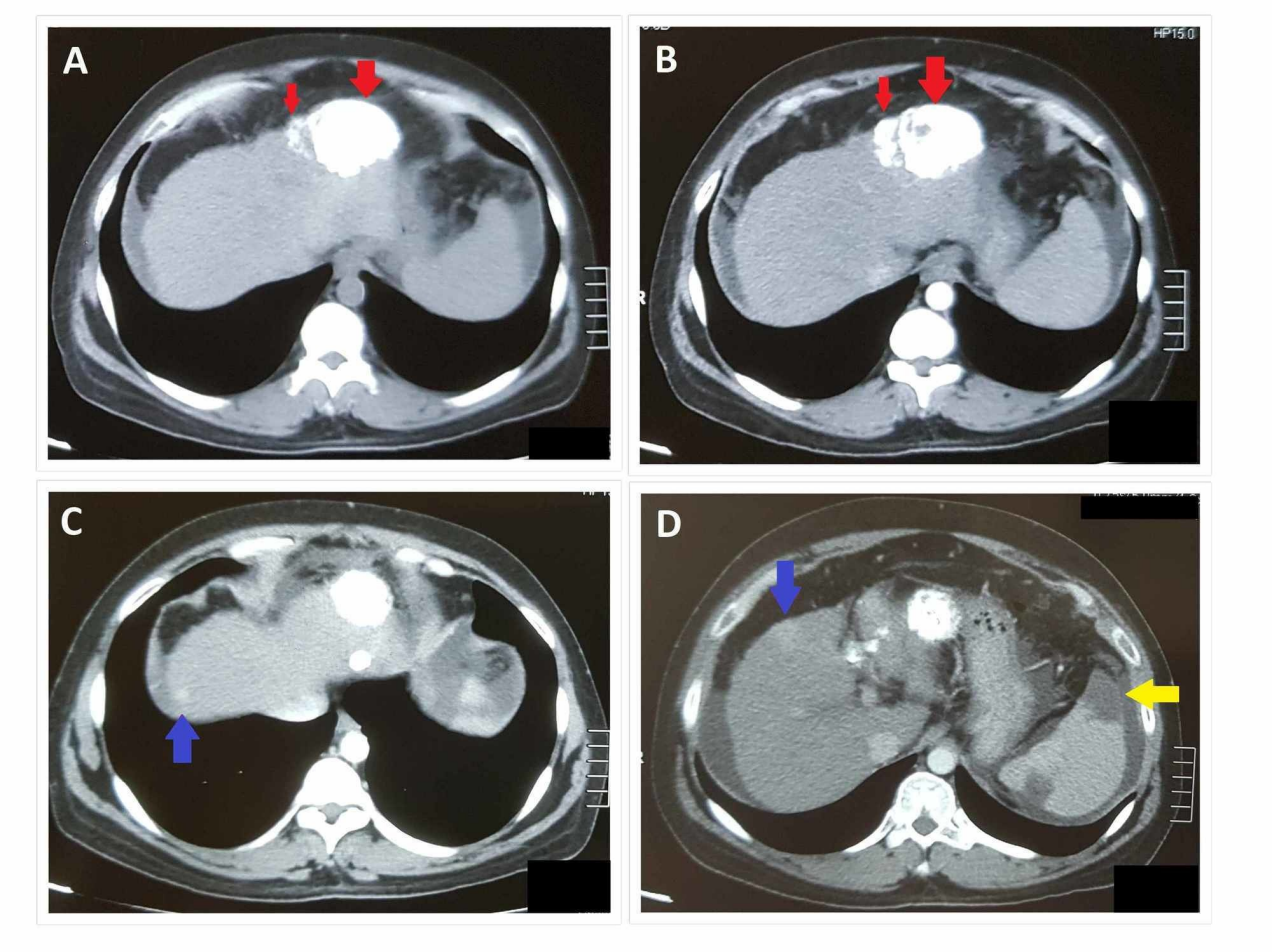
Triphasic CT abdomen at one-month follow-up after TACE showing two areas of dense hyperdensities (red arrows in A and B) in the left lobe of the liver at site of previous HCC representing lipoidal deposition (status post TACE) There is a new development of two small arterially enhancing lesions in the right lobe of the liver (blue arrows in C and D), suggestive of hepatomas. Spleen appears mildly enlarged. Wedge-shaped peripheral hypodense areas (yellow arrow) are seen within the splenic parenchyma suggestive of splenic infarcts. CT: computed tomography; TACE: transarterial chemoembolization; HCC: hepatocellular carcinoma

The patient underwent successful chemoembolization in April 2019 and was started on Tablet Tenofovir 300 mg once daily with advice to abstain from alcohol. The patient remained well after that. At one-month follow-up, CT abdomen was performed, which showed post-TACE radiological changes in left lobe of the liver (Figure 2) and development of two new small-sized lesions in the right lobe of liver consistent with hepatomas. No change in treatment was done and the patient was called for follow-up after one month.

In July 2019, the patient presented to the Gastroenterology & Hepatology Department, Nishtar Hospital, Multan, Pakistan with complaints of jaundice and abdominal distension from the last two weeks. He also complained of right hypochondrium pain associated with nausea and vomiting. There was no history of fever. There was a history of passing black tarry stools for the last few days. The patient also admitted that he started drinking alcohol again after abstinence of a few weeks. No history of illicit drug intake. Examination showed pallor, palmar erythema, and yellow sclera. Flapping tremors were absent. The abdomen was soft with mild tenderness in right hypochondrium and there was distension with fullness in flanks. The liver was enlarged, hard, and tender with irregular margins. There was evidence of ascites and splenomegaly.

**Figure 3:**
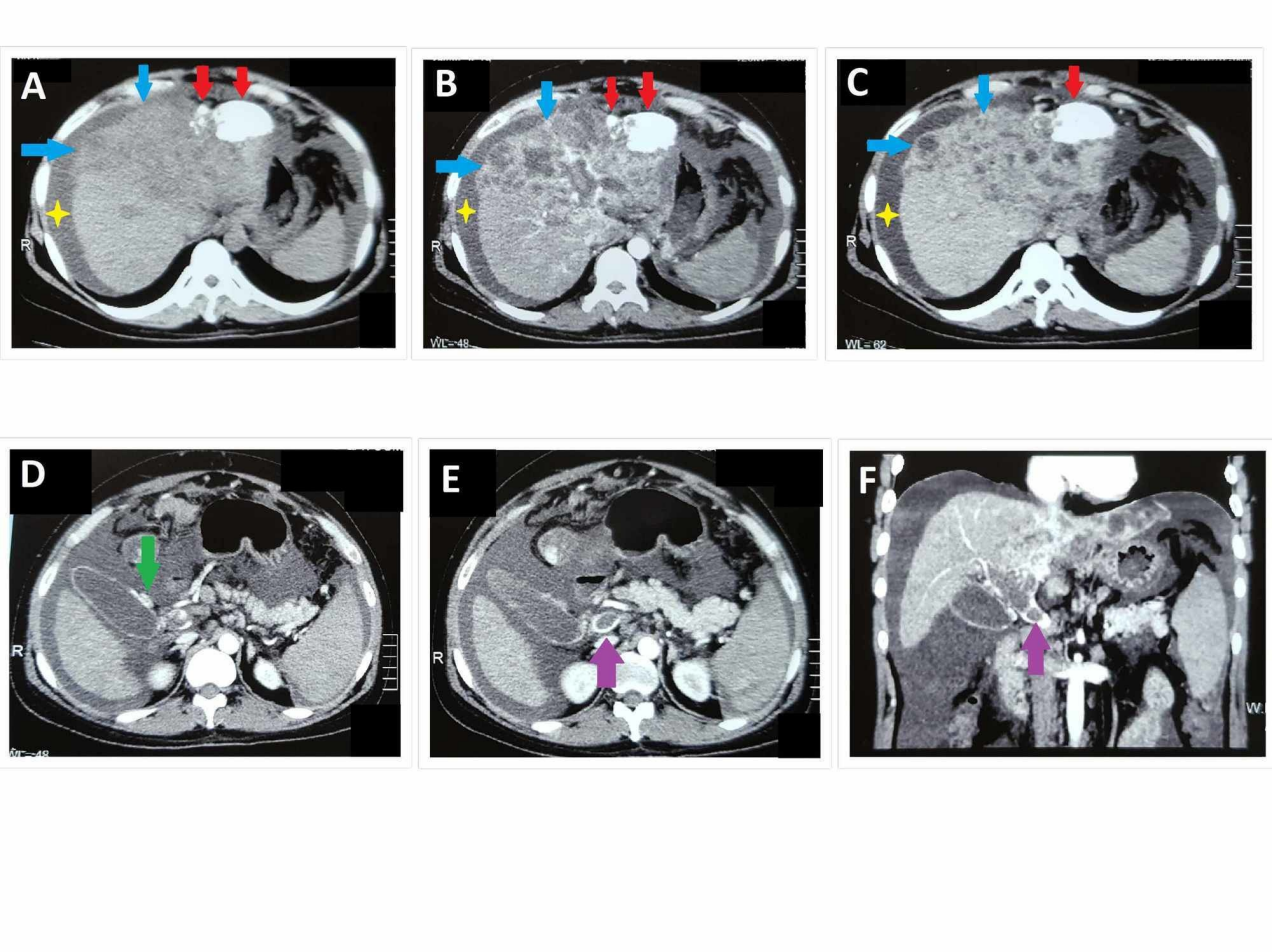
Two months post-TACE triphasic CT abdomen showing plain (A), arterial (B), and venous (C) phases of the study Two areas of dense hyper-densities (red arrows) are identified in the left lobe of liver-post TACE changes. There are newly developed multiple ill-defined infiltrative lesions (blue arrows) involving the left and right lobes of liver showing arterial hyperenhancement (B) and washout on venous phases (C). The liver has irregular margins and nodular contour, and there are moderate abdominopelvic ascites (yellow star). Dilated vascular channels are noted within the wall of the gallbladder (D) representing the varices formation (green arrow). These are showing arterial filling suggestive of AV shunting due to primary hepatic malignancy. Portal vein (purple arrow) is dilated showing arterial hyperenhancement along with a filling defect, suggestive of AV shunting and portal vein thrombosis. Peripancreatic lymph nodes are also seen. CT: computed tomography; TACE: transarterial chemoembolization; AV shunting: arteriovenous shunting

Ultrasound abdomen showed coarse echotexture of the liver. There were multifocal hypoechoic lesions seen in left and right lobes of the liver. Triphasic CT scan abdomen showed newly formed multiple infiltrative lesions suggestive of HCC in both lobes of the liver (Figure 3), portal vein thrombosis, arteriovenous shunting, and moderate ascites.

Laboratory investigations are shown in Table 1. Ascitic fluid routine examination showed high serum ascites albumin gradient (SAAG) ascites with no evidence of spontaneous bacterial peritonitis (SBP). The patient was managed with intravenous antibiotics, dextrose, vasopressors and syrup lactulose. Esophagogastroduodenoscopy (EGD) showed early esophageal varices and portal gastropathy. A diagnosis of acute on chronic liver disease due to the rapid intrahepatic progression of HCC was made. His current child class is C, model for end-stage liver disease (MELD) score 20, performance status 1-2, and BCLC stage D. After a few days of treatment, the patient gradually improved and was discharged on diuretics, beta-blockers and lactulose. He was called for follow-up after two weeks.

**Table 1:** Laboratory investigations WBC: white blood cells; RBC: red blood cells; ALT: alanine aminotransferase; AST: aspartate aminotransferase; ALP; alkaline phosphatase; HBsAg: surface antigen of hepatitis B virus; Anti-HCV: antibody to hepatitis C virus; HBeAg: e antigen of hepatitis B virus; Anti HIV: antibodies to human immunodeficiency virus; Anti-HDV: antibodies to delta virus; Anti HAV IgM: antibodies to hepatitis A virus IgM subtype; Anti HEV IgM: antibodies to hepatitis E virus IgM subtype; INR: international normalized ratio; AFP: alpha-fetoprotein

Peripheral Blood	Results	Blood Chemistry	Results
WBC	14000 /µL	Serum Bilirubin	4.8 mg/dl
Neutrophils	85 %	ALT	95 U/L
Lymphocyte	10 %	AST	44 U/L
Mixed	5 %	ALP	582 U/L
		Serum Albumin	2.0 g/dl
RBC	2.660x 10^6^ / µL	Urea	45 mg/dl
Hemoglobin	8.5 g/dl	Creatinine	0.7 mg/dl
Hematocrit	26.4 %	Sodium	136 mmol/L
Platelet count	67000 /µL	Potassium	4.54 mmol/L
Coagulation Test		Serological Tests	
Prothrombin Time (PT)		HBsAg	Positive
Patient PT	24 seconds	Anti-HCV	Negative
Control (PT)	12 seconds	HBeAg	Positive
INR	2	Anti HIV	Negative
		Anti-HDV	Negative
Tumor Marker		Anti HAV IgM	Negative
AFP Level	968 ng/ml	Anti HEV IgM	Negative

## Discussion

TACE is a good alternative for HCC patients who are not suitable for surgical resection and have no vascular invasion and no metastasis. The association of TACE and the rapid progression of HCC has been discussed in different studies. In an experimental study by Wang GZ et al, HCC was induced in buffalo rats. It demonstrated statistically significant increased metastasis in the transarterial embolization group. It was proposed that hypoxia leads to an increase in a transcriptional factor called Hypoxia-inducible factor-1 (HIF-1) which plays an important role in the breakthrough of the tumor by damaging the extracellular matrix and intracellular adhesion molecules. Other mechanisms like the formation of new blood vessels in response to hypoxia may also play some role [3]. These results were validated by Lai JP et al. study. This, in addition to previous findings, showed that hypoxia is also related to the activation of proteins that are involved in promoting the differentiation of progenitor cells. These factors may result in the manifestation of aggressive behavior by the tumor following TACE [4].

There are multiple studies highlighting the aggressive recurrence of tumor following radiofrequency ablation (RFA), but not many studies could be found in the literature regarding similar association with TACE [5-6]. A retrospective study was conducted to evaluate the frequency of rapid recurrence of HCC when a combination of TACE and RFA was used. Interestingly, the results showed a decline in the overall occurrence of this complication when combined therapy was used [7]. Our patient underwent TACE only and developed features of acute liver failure within a few months of intervention. A study aimed to describe the adverse effects of TACE, demonstrated the complication of acute decompensation in a few cases [2]. In our patient the underlying pathogenesis of rapid intrahepatic progression of tumor leading to acute, chronic disease was unique. This is a rare occurrence following TACE.

Our patient was managed on the lines of acute on chronic liver disease. He was discharged after improvement in symptoms and was called for follow-up after two weeks. In light of this, it is imperative for physicians to discuss this adverse effect with patients who are planning to go for TACE. Further research is required for a deeper understanding of pathogenesis and factors responsible for this complication.

## Conclusions

The rapid progression of primary liver tumor in response to TACE therapy is a rare complication. Hypoxia induced by this intervention is considered to play an important role in its manifestation. Herein, we present a case of an HCC patient who developed acute liver failure within a few months after TACE procedure. On triphasic CT scan, multiple new infiltrative lesions suggesting HCC were found, involving both lobes of the liver. This is a unique case of rapid HCC progression following TACE therapy. 
